# Pharmacological ablation of astrocytes reduces Aβ degradation and synaptic connectivity in an ex vivo model of Alzheimer’s disease

**DOI:** 10.1186/s12974-021-02117-y

**Published:** 2021-03-17

**Authors:** Nicola Davis, Bibiana C. Mota, Larissa Stead, Emily O. C. Palmer, Laura Lombardero, Rafael Rodríguez-Puertas, Vincenzo de Paola, Samuel J. Barnes, Magdalena Sastre

**Affiliations:** 1 Department of Brain Sciences, Imperial College London, Hammersmith Hospital, London, W12 0NN UK; 2 Department of Pharmacology, University of the Basque Country (UPV/EHU), 48940 Leioa, Spain; 3 Institute of Clinical Sciences, Imperial College London, London, W12 0NN UK; 4 Imperial College UK-Dementia Research Institute, Hammersmith Hospital, London, W12 0NN UK

**Keywords:** Astrocytes, L-AAA, Cytokines, Astrocytes, Amyloid-β, Synapsis, Organotypic cultures

## Abstract

**Background:**

Astrocytes provide a vital support to neurons in normal and pathological conditions. In Alzheimer’s disease (AD) brains, reactive astrocytes have been found surrounding amyloid plaques, forming an astrocytic scar. However, their role and potential mechanisms whereby they affect neuroinflammation, amyloid pathology, and synaptic density in AD remain unclear.

**Methods:**

To explore the role of astrocytes on Aβ pathology and neuroinflammatory markers, we pharmacologically ablated them in organotypic brain culture slices (OBCSs) from 5XFAD mouse model of AD and wild-type (WT) littermates with the selective astrocytic toxin L-alpha-aminoadipate (L-AAA). To examine the effects on synaptic circuitry, we measured dendritic spine number and size in OBCSs from Thy-1-GFP transgenic mice incubated with synthetic Aβ42 or double transgenics Thy-1-GFP/5XFAD mice treated with LAAA or vehicle for 24 h.

**Results:**

Treatment of OBCSs with L-AAA resulted in an increased expression of pro-inflammatory cytokine IL-6 in conditioned media of WTs and 5XFAD slices, associated with changes in microglia morphology but not in density. The profile of inflammatory markers following astrocytic loss was different in WT and transgenic cultures, showing reductions in inflammatory mediators produced in astrocytes only in WT sections. In addition, pharmacological ablation of astrocytes led to an increase in Aβ levels in homogenates of OBCS from 5XFAD mice compared with vehicle controls, with reduced enzymatic degradation of Aβ due to lower neprilysin and insulin-degrading enzyme (IDE) expression. Furthermore, OBSCs from wild-type mice treated with L-AAA and synthetic amyloid presented 56% higher levels of Aβ in culture media compared to sections treated with Aβ alone, concomitant with reduced expression of IDE in culture medium, suggesting that astrocytes contribute to Aβ clearance and degradation. Quantification of hippocampal dendritic spines revealed a reduction in their density following L-AAA treatment in all groups analyzed. In addition, pharmacological ablation of astrocytes resulted in a decrease in spine size in 5XFAD OBCSs but not in OBCSs from WT treated with synthetic Aβ compared to vehicle control.

**Conclusions:**

Astrocytes play a protective role in AD by aiding Aβ clearance and supporting synaptic plasticity.

**Supplementary Information:**

The online version contains supplementary material available at 10.1186/s12974-021-02117-y.

## Background

Astrocytes comprise 20% of the cells in the brain [1] and play multiple roles in brain homeostasis. Their functions include responding to CNS insults [2], providing metabolic support (production of cholesterol and lactate), regulating of the brain’s inflammatory response [1], and performing supportive functions such as maintaining the extracellular pH and ion content and aiding in the recycling of neurotransmitters such as glutamate and removal of waste products [3]. When an insult occurs, astrocytes become reactive and undergo astrogliosis [2]. This causes a change in their morphology, becoming hypertrophic, as their cell body enlarges, and their processes grow and develop into thick branches [4]. The presence of astrogliosis is a classic feature of AD [1], and reactive astrocytes are known to cluster around Aβ plaques, forming a glial scar [5–7]. However, it is unclear the extent to which astrocytes may change their supportive functions when they become reactive [3].

Whether reactive astrocytes have a beneficial or detrimental role in AD is debated [8]. They can cause damage as they play a part in the perpetuation of chronic inflammation [9]. They produce pro-inflammatory mediators, such as nitric oxide [8], thus increasing neuroinflammation and the associated damage [1]. In addition, they increase the levels of reactive oxygen species (ROS) and produce hydrogen peroxide causing oxidative stress [1]. While in the short term, this is advantageous as they provide protection against insults, and the chronic activation of astrocytes may become damaging. In addition, astrocytes may be involved in the generation of Aβ as there is evidence that under inflammatory conditions, they can express the components required for Aβ generation, i.e., APP, BACE1, and γ-secretase [10, 11].

On the other hand, reactive astrocytes can be neuroprotective in the AD brain. They form a fibrous scar, which helps to contain inflammation, and provide trophic support to neurons by expressing neurotrophic factors and regulate synaptic formation [5, 12]. In addition, we and others have reported that reactive astrocytes are involved in the degradation and clearance of Aβ [13, 14]. Astrocytes can produce Aβ peptidases, such as neprilysin and IDE as well as the Apolipoprotein E (ApoE) [15], which binds to and is involved in the clearance of Aβ [16]. The use of genetic depletion of reactive astrocytes has allowed the investigation into their role in vivo. We have recently shown that the loss of proliferating astrocytes leads to increased amyloid levels and exacerbated memory loss in the APP23 model of AD [17].

Besides using genetic models, there are other potential strategies to determine the effect of astrocytic loss in AD. One of them is the pharmacological ablation of astrocytes by incubating them with the glutamate analogue L-alpha-aminoadipic acid (L-AAA), which causes selective astrocytic toxicity and death [18]. This drug binds to the cysteine glutamate antiporter, which is expressed in astrocytes, but not in microglia or neurons, allowing it to selectively target astrocytes. Because this drug does not cross the BBB, we sought to investigate the effect of pharmacological ablation of astrocytes in organotypic cultures. The process of organotypic culture involves culturing complete slices of brain tissue on semipermeable membranes. This technique allows the study of the brain in its original structure [19, 20]. Our aim was to determine the role of pharmacological ablation of astrocytes using organotypic cultures of the 5XFAD model of AD on neuroinflammatory markers, amyloid pathology, and dendritic spine density.

## Methods

### Materials

The antibodies used for detection of proteins of interest were 6E10 (against Aβ1-16) from Covance, R1(57) against the carboxy terminus of APP was a kind gift from Dr P. Mehta (NYS Institute for Basic Research in Developmental Disabilities); anti-BACE1 was from Cell Signaling; anti-apolipoprotein E (ApoE) and anti-neprilysin CD10 from Santa Cruz; anti-ionized calcium-binding adapter molecule 1 (IBA1) from Wako; anti-glial fibrillary acidic protein (GFAP) (clone 2.2B10), anti-Aβ (6C3), and anti-neuronal nuclei (NeuN) were from Millipore; and anti-insulin degrading enzyme (IDE), Aldehyde dehydrogenase 1A (Aldh1a1), and anti-β-actin were from Abcam. Tissue culture reagents were purchased from Invitrogen and Millipore, and all other reagents were purchased from Sigma, unless stated otherwise.

### Animal models

5XFAD transgenic (Jackson Laboratory) 7-day-old pups were used for organotypic cultures, overexpressing in their brains human APP (695) with the Swedish (K670N, M671L), Florida (I716V), and London (V717I) mutations, as well as human PS1 with the mutations M146L and L286V [21]. 5XFAD mice were used because their phenotype is more aggressive than other transgenic models of amyloidosis and allow the detection of pathological changes in brain slices after only 2 weeks in culture. Thy-1-GFP transgenic 7-day-old pups were used for organotypic cultures, expressing membrane-bound GFP [22].

Mice were kept in individually ventilated cages and maintained on a 12/12-h light/dark cycle with controlled temperature and humidity, and food and water ad libitum. In vivo procedures (breeding) were performed in accordance to the United Kingdom Animal (Scientific Procedures) Act (1986) and approved by Imperial College London’s Animal Welfare and Ethical Review Body.

### Organotypic brain cultures

Organotypic brain culture slices (OBCSs) were prepared from postnatal day (P) 7 from wild-type, 5XFAD, Thy-1-GFP, and double transgenics Thy-1-GFP/5XFAD mice. The brains were sectioned in 300-μm coronal slices using a vibratome (Leica VT1200 S); sections were then mounted on semi-porous membrane filters (Millipore). Brain slices were maintained in culture for 2 weeks in nutrient media (Neurobasal-A medium (Invitrogen), 20% normal horse serum, 20% HBSS, 0.5% glutamine, 0.5% vitamin B27 supplement, and 1% antibiotics) and incubated at 37 °C, 95%HR, 5%CO_2_. The nutrient medium was replaced every 2 days for 2 weeks. Slices from 5XFAD mice were treated for 24 h with vehicle or with 1 mM L-AAA (Sigma) [18] in nutrient media. In another set of experiments, WT OBSCs were treated with vehicle or 0.6 μM synthetic Aβ42 [23] (Anaspect). After incubation, media was collected and brain slices were either homogenized or fixed in 4% PFA for 24 h and kept at 4 °C in PBS and Na-azide.

### Western blot

Brain slices were homogenized in RIPA buffer (1% Triton X-100, 1% sodium deoxycholate, 0.1% SDS, 150 mM NaCl, and 50 mM Tris-HCl, pH 7.2) supplemented with cOmplete protease inhibitor (Roche) and phosphatase inhibitor (Roche). A 50 μg of protein either from homogenates or conditioned media were run on 4–12% Tris-tricine gels (NuPAGE gels, Invitrogen). After transferring the gel to PVDF or nitrocellulose (for Aβ) membranes, the membranes were blocked with 5% non-fat semi skimmed milk diluted in Tris-buffered saline with Tween (TBST) for 1 h. The primary antibody, diluted in 1% BSA and Na-azide, was subsequently incubated overnight at 4 °C. The horseradish peroxidase secondary antibody was incubated in 5% non-fat semi-skimmed milk for 1 h at room temperature and bands were visualized using ECL with a Gengnome XRQ device. Western blots were analyzed using the ImageJ software and values were normalized using β-actin as loading control.

### Enzyme-linked immunosorbent assays (ELISA)

The levels of human Aβ40 and Aβ42 were determined in homogenates using the High Sensitivity Human Amyloid β42 and Aβ40 ELISA kits from Millipore. For the analysis of mouse cytokines and chemokines in conditioned media from the cultures, we used kits from Peprotech (IL-1β, TNFα and IL-4) and Meso Scale Discovery (Europe) (IL-6, IL-10, TGF-β1, MCP-1, and MIP-1α). Concentrations were quantified according to the manufacturer’s instructions and normalized to total protein concentration.

### RNA extraction and quantitative PCR

mRNA extraction was conducted using the mirVana™ microRNA (miRNA) Isolation Kit according to manufacturer instructions (Thermofisher). Organotypic slices were homogenized in Precellys tubes using a MiniLys homogenizer (Bertin Technologies).

Polymerase chain reaction real-time cycling was carried out with PowerUp™ SYBR™ Green Master Mix (Thermofisher) and Quantitect Primer assays (Qiagen) for mouse Neprilysin (Mme, Qiagen), IDE (5′-CAGAAGGACCTCAAGAATGGGT-3′ and GCCTCGTGGTCTCTCTTTATCT) and ApoE (Primers: ′-GGGACAGGGGGAGTCCTATAA-3′ and 5′-ATTGGCCAGTCAGCTCCTTC-3′) and normalized to GAPDH (5′-ACCACAGTCCATGCCATCAC-3′ and 5′-TCCACCACCCTGTTGCTGTA-3′). A 2-step method with an initial reverse transcription and subsequent real-time cycling on an Aria Mx qPCR workstation cycler was performed, as reported previously [17].

### Propidium iodide (PI) staining

PI was added at a concentration of 5 μg/ml, incubated for 30 min in the conditioned media, and imaged with a confocal microscope.

### Immunofluorescence staining

Brain sections were permeabilized overnight in 0.25% TBS-TX (TBS with triton X-100). Following this, sections were washed again in TBS and blocked for 60 min in 10% goat serum/1%BSA in 0.1% TBS-TritonX and incubated with the primary antibodies (anti-GFAP, 1:500, anti-NeuN 1:500, anti-IBA1 1:500, anti-ALDH1L1 1:500, anti-6C3 1:500) diluted in 2% goat serum, 0.2% BSA in TBS-TX 0.02% for 48 h at 4 °C. Following this, sections were washed 5 times for 10 min in TBS and incubated with the secondary fluorescent antibodies (1:400 Alexa Fluor; Invitrogen) in 2% goat serum, 0.2% BSA in TBS-TX 0.02% overnight at 4 °C. After incubation with the secondary antibodies, sections were washed 4 times with TBS for 10 min. In the final wash, Hoechst solution (for nuclear visualisation) was added (1:1000 in TBS) for 5 min. Following this, the slices were washed and mounted using ProlongTM Gold antifade reagent (Invitrogen). Sections were visualized with a confocal microscope (Zeiss LSM-780). Immunohistochemistry staining was quantified using the HALO software (Indico Labs) using the area quantification FL module and represented as percentage of the total image area.

### Quantification of dendritic spines and analysis of microglia

#### Dendritic spine size and density

Spine size was calculated as integrated brightness, using an adapted version of custom written MATLAB code, as described previously [24, 25]. Briefly, the spine intensity was measured by first drawing a region of interest (ImageJ) across a length of dendrite taken from confocal *z*-stacks. The region of interest covered the spine protrusions and a section of the adjacent dendrite. The region of interest was then used to measure the fluorescence intensity profile of the spines and adjacent dendrite. The background was subtracted, and the intensity profile was normalized to the adjacent dendrite to account for differences in image intensity and background noise between acquired *z*-stacks using MATLAB. A custom written peak detection code (MATLAB) was then used to identify fluorescence peaks in the normalized intensity profile corresponding to individual spines. To identify peaks, a threshold (50% greater than adjacent dendritic fluorescence) was set based on visual inspection of the smallest detectable spine peak. The number of spine peaks was then divided by the length of the dendrite to estimate the spine density and confirmed by manually counting spines along 3-dimensional dendritic path-lengths using ImageJ. For each spine, the area under the normalized fluorescence spine peak (measured in normalized dendrite units) was used as a proxy for spine size.

#### Microglia analysis

For microglia analysis, a fluorescence intensity profile, taken from a maximum intensity projection, was drawn through the longest dimension of the cell body and across a cross-section of the outer most tips of all associated processes. This intensity profile was then used to estimate the soma size as well as the number, area, and the perimeter of processes using custom written code in MATLAB. Soma size was estimated by normalizing the portion of the fluorescence trace corresponding to the soma to the background and then calculating the area under the curve for this section by multiplying the fluorescence peak by the width. The number of branch processes was estimated by using custom written code (MATALB) to detect peaks that were 25% greater than background (threshold derived from visual inspection). These peaks were then used to estimate the process area by calculating the area under each fluorescence peak and summing these values. Finally, the total process perimeter was calculated by adding the total number of pixels covered by all detected peaks and dividing this number by the scaling factor or the image (pixels/μm).

### Statistical analysis

We calculated the number of animals and group sizes to be used via InVivoStat, an R-based statistical package [26]. The data was analyzed using Graphpad Prism 8 software (Graphpad Software Inc), using unpaired Student’s *t* tests, repeated-measures 1- or 2-way analyses of variance, and followed by Tukey post hoc analysis. The Kolmogorov-Smirnov test was applied to confirm normal distribution. All quantitative data are given as mean ± SEM. Probability of less than 0.05 was considered significant.

## Results

### L-AAA treatment results in astrocytic death in organotypic brain cultures

**Fig. 1:**
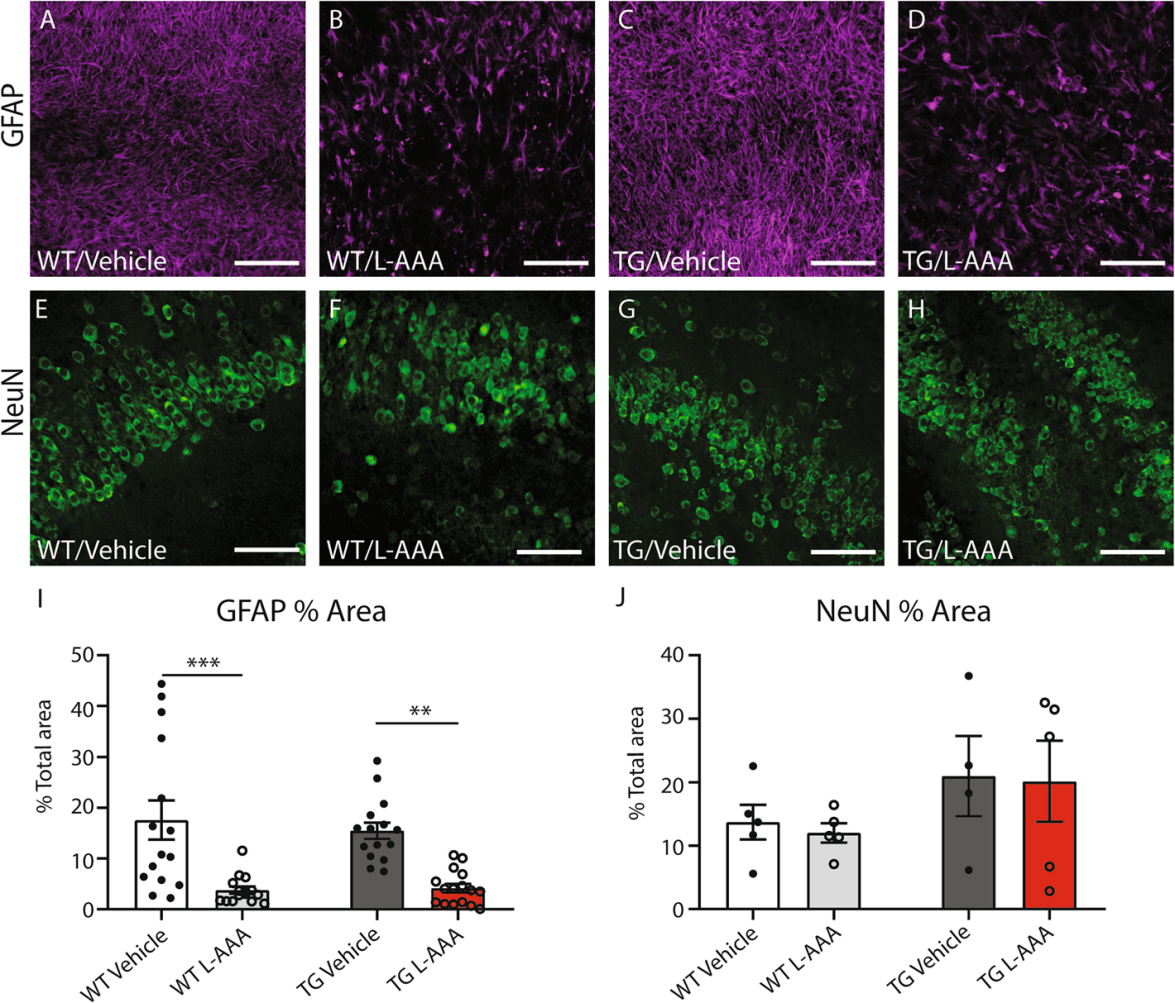
L-AAA reduces the density of astrocytes but does not affect the number of neurons. Representative images and quantification of astrocytes and neurons in OBCSs treated with 1mM L-AAA or vehicle for 24 h. **a**–**d** GFAP expression in the hippocampus of OBCSs from WT and 5XFAD mice treated with 1mM L-AAA or vehicle for 24 h. **e**–**h** Expression of NeuN in the hippocampus of OBCSs from WT and 5XFAD mice. **i** Quantification of % area stained with GFAP (*n*=15–16). **j** Quantification of % area stained with NeuN (*n*=4–5). Values shown in graphs represent the mean value ± SEM. Statistical analysis included a Student’s *t* test, **P<0.01 ***P<0.001. Scale bar = 100 μM

To determine the effectiveness of the L-AAA treatment on selective astrocytic toxicity and death [18, 27], sections were incubated at two different time points and stained with GFAP and Aldehyde dehydrogenase 1A (Aldh1a1) for astrocytes and NeuN antibodies for neurons. Our results show a remarkable reduction on GFAP and Aldh1a1 staining after 24 h treatment (Fig. 1a–d, Fig. S1A-F), without affecting the viability and number of neurones (Fig. 1e–h and j). However, at 48 h, tissue organization was largely disrupted (Fig. S1G). To prove that at 24 h the sections were not damaged, slices were stained with propidium iodide (PI), demonstrating that the incubation with L-AAA for 24 h did not affect tissue integrity (Fig. S1H-K). Therefore, 24 h was selected as the optimal L-AAA length of exposure and was used in the following experiments.

### Pharmacological ablation of astrocytes leads to an increase in the expression of pro-inflammatory markers

**Fig. 2:**
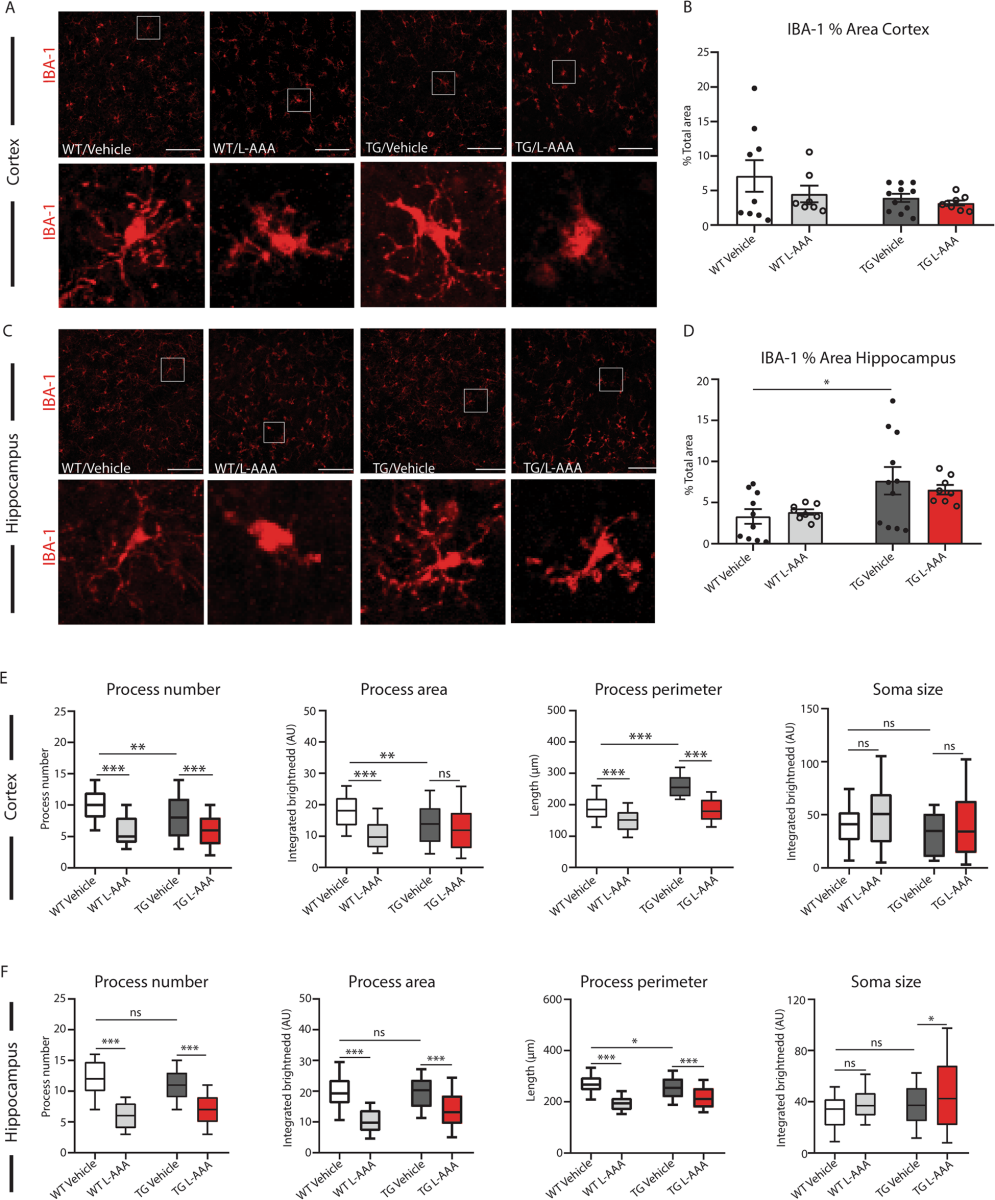
L-AAA treatment affects the morphology of microglia in the cortex and hippocampus. **a** Representative images and **b** quantification of the % area of Iba-1 staining in cortex of WT and 5XFAD OBCSs treated with L-AAA for 24h (*n*=7–9). **c** Representative images and **d** quantification of the % area of Iba-1 staining in hippocampus of WT and 5XFAD OBCSs treated with L-AAA for 24 h. Scale bar=100 μM (*n*=8-11). **e** Analysis of process number, process area, process perimeter, and soma size of Iba-1-positive microglia in the cortex (**e**) and hippocampus (**f**) (*n*=7–11). Values shown in graphs represent the mean value ± SEM. Statistical analysis included a one-way ANOVA, *P<0.05; **P<0.01; ***P<0.001

We next examined whether the loss of astrocytes affected the density of other glial cells, such as microglia. The staining of microglia with Iba-1 antibody in cortex and hippocampus (Fig. 2a–d) showed an increase in the density of microglia in sections from 5XFAD mice compared with WTs, which was significant in the hippocampus (Fig. 2d). However, it did not reveal changes in the density of microglia in sections of 5XFAD mice treated with 1 mM L-AAA compared with vehicle treated controls (Fig. 2a–d). However, high magnification images showed a change in the morphology of microglia, being more amoeboid-like and less ramified in transgenic OBCSs and in all sections following treatment with L-AAA, indicative of an activated phenotype (Fig. 2). Analysis of the morphological parameters of microglia demonstrated a reduction in the number of processes, in the process area and the process perimeter of slices treated with L-AAA (Fig. 2e, Fig. 2f), while the soma size was increased, particularly in microglia of the transgenic hippocampus, typical of a reactive state (Fig. 2f)

**Fig. 3:**
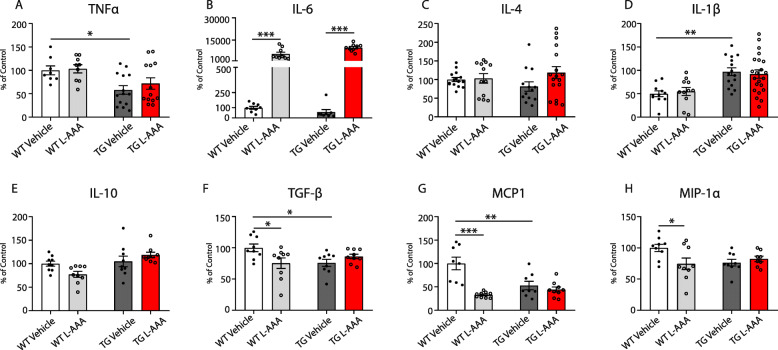
L-AAA treatment affects the neuroinflammatory profile in the cortex. Quantification of expression of **a** TNFα, **b** IL-6, **c** IL-4, **d** IL-1β, **e** IL-10, **f** TGF-β, **g** MCP1, and **h** MIP-1α by ELISA in conditioned media from OBCSs of WT and 5XFAD mice treated with L-AAA for 24 h (*n*=6–18). Values shown in graphs represent the mean value ± SEM. Statistical analysis included one-way ANOVA with Tukey’s multiple-comparison post-hoc test. *P<0.05; **P<0.01; ***P<0.001

Following this, we measured the levels of various pro- and anti-inflammatory cytokines and chemokines in the conditioned media of the organotypic cultures (Fig. 3). Interestingly, media from transgenic 5XFAD slices showed different cytokine profile, with higher Il-1β levels and reduced TNFα, TGFβ, and MCP1 expression, compared with WT sections, in line with the changes found on microglia morphology (Fig. 3a, d, f, and g). In addition, we found that the expression of pro-inflammatory cytokine IL-6 was increased in both WT and transgenic sections treated with L-AAA (Fig. 3b), while other cytokines and chemokines mostly produced by astrocytes, such as TGF-β1, MCP1(CCL2), and MIP-1α (CCL3) [28], were reduced by incubation with L-AAA only in wild-type sections (Fig. 3f–h). IL-1β, IL-10, and IL-4 levels were unchanged by L-AAA treatment in all groups analyzed (Fig. 3c–e).

These results suggest that depletion of astrocytes results in an increase of the pro-inflammatory profile in organotypic brain cultures.

### L-AAA treatment affects Aβ degradation mechanisms

**Fig. 4:**
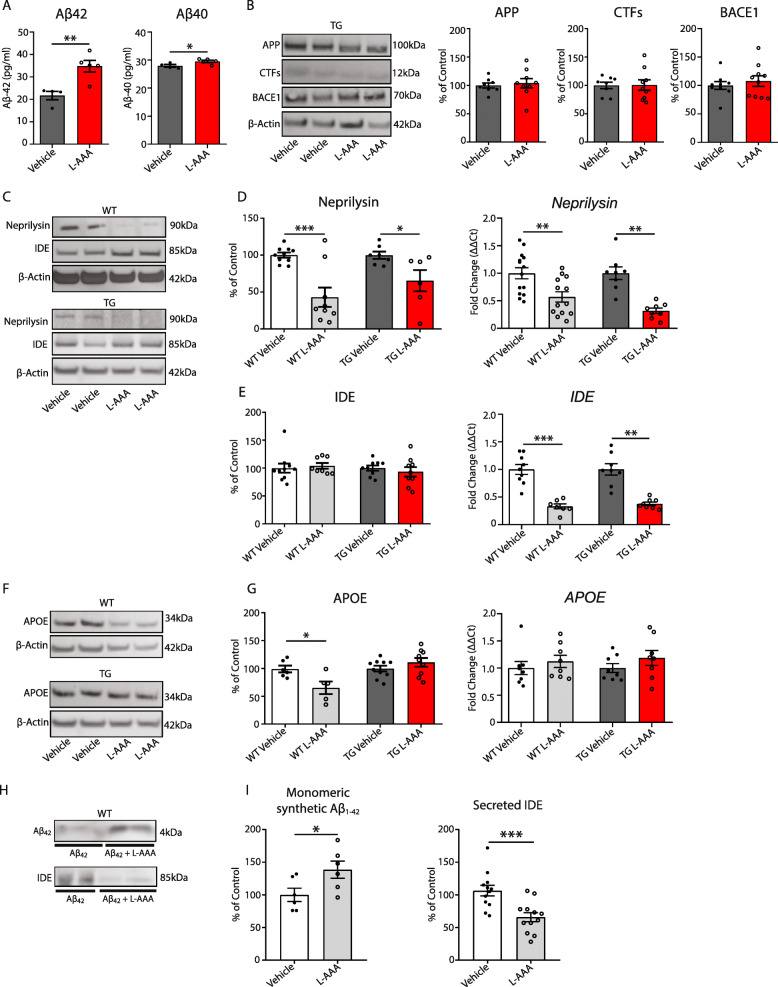
Loss of astrocytes affects Aβ clearance mechanisms. **a** Quantification of Aβ subtypes by ELISA in 5XFAD OBCSs homogenates (*n*=4–5). **b** Quantification and representative Western blot of APP, CTFs, and BACE1 expression in 5XFAD OBCS homogenates (*n*=6–10). **c** Representative Western blot of Neprilysin and IDE protein expression in WT and 5XFAD treated with vehicle or L-AAA. **d** Quantification of Neprilysin protein and gene expression in WT and 5XFAD treated with vehicle or L-AAA (*n*=6–12). **e** Quantification of IDE protein and gene expression in WT and 5XFAD OBCSs treated with vehicle or L-AAA (*n*=6–12). **f** Representative Western blots and **g** quantification of ApoE protein and gene expression in WT and 5XFAD OBCSs OBCSs treated with vehicle or L-AAA (*n*=5–10). **h** Representative Western blot of synthetic human Aβ1-42 and IDE in conditioned media from WT OBCSs treated for 24h with L-AAA or vehicle **i** quantification of Aβ42 by Western blot (*n*=12–18) and quantification of IDE protein expression in conditioned media from WT OBCSs treated for 24 h with synthetic human Aβ1-42 with vehicle or L-AAA (*n*=12–18). Values shown in graphs represent the mean value ± SEM. Statistical analysis included a Student’s *t* test, *P<0.05; **P<0.01; ***P<0.001

To investigate the effects of pharmacological loss of astrocytes on amyloid-β levels, transgenic sections treated with L-AAA overnight were homogenized and the expression of Aβ subtypes was measured by ELISA. At this age, cultures do not show Aβ deposition in plaques (Fig. S1M). Our results show that L-AAA treatment led to a 65% increase in the levels of Aβ1-42, while the expression of Aβ1-40 was only slightly increased compared with vehicle controls (Fig. 4a).

To determine the potential mechanisms leading to the increase on Aβ expression, we measured the levels of a full-length APP and the CTFs in OBCS homogenates. The expression of full-length APP and the CTFs were found unaltered after L-AAA treatment (Fig. 4b), suggesting that the cleavage of APP was not affected by the loss of astrocytes. In agreement with these results, the expression of the β-secretase (BACE1) did not change either in any of the groups analysed (Fig. 4b).

We next analyzed whether the mechanisms of clearance and degradation of Aβ were affected by L-AAA, since we have previously observed that they were altered in a model of genetic ablation of astrocytes [17]. In agreement with our previous report, we found that L-AAA induced a significant reduction of the protein and mRNA levels of neprilysin (Fig. 4c, d) and insulin-degrading enzyme (*Ide*) gene expression (Fig. 4c, e). ApoE protein levels were only reduced in WT sections (Fig. 4f, g).

To confirm that the loss of astrocytes affects the mechanisms of Aβ clearance and degradation, we treated wild-type slices with 0.6-μM synthetic Aβ1-42, in the presence or absence of 1 mM L-AAA for 24 h, to allow the measurement of the stability of Aβ. Analysis of Aβ in culture media by Western blot revealed higher levels of Aβ in L-AAA treated sections (>56%) compared with vehicle-treated control (Fig. 4h, i). In addition, the expression levels of IDE were significantly reduced in the media of cultures treated with L-AAA (Fig. 4h, i). These data indicate that the stability of synthetic Aβ1-42 peptide in the medium was increased by incubation with L-AAA, due to reduced Aβ degradation.

### Pharmacological ablation of astrocytes leads to dendritic spine loss

**Fig. 5:**
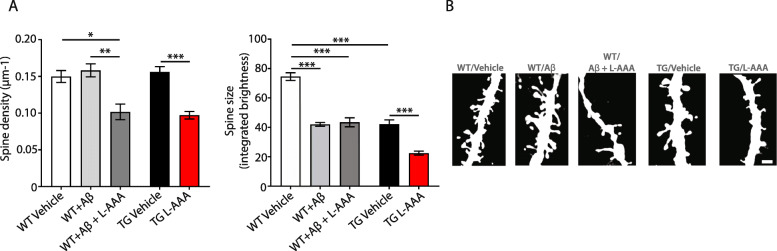
Ablation of astrocytes alters synaptic density in OBCSs. (**a**) Quantification and (**b**) representative images of dendritic spine density and size in hippocampus (CA1 and CA3 areas) of organotypic cultures of thy-1-GFP transgenic mice crossed with 5XFAD mice or WTs incubated with 0.6-μM synthetic Aβ and treated for 24 h with L-AAA or vehicle (*n*=12–18). Scale bar= 1 µm values shown in graphs represent the mean value ± SEM. Statistical analysis included one-way or two-way ANOVA with Tukey’s or Holm-Sidaks multiple-comparison post-hoc test, *P<0.05; **P<0.01; ***P<0.001

To examine the effects of pharmacological ablation of astrocytes on synaptic connectivity, we measured the number and size of dendritic spines in organotypic cultures from brains of thy-1-GFP transgenic mice incubated with 0.6-μM synthetic Aβ1-42 or crossed with 5XFAD mice. Quantification of dendritic spines revealed a 33% reduction in their density in the CA1 area of the hippocampus in all groups following L-AAA treatment (Fig. 5a, b).

Interestingly, spine size was reduced in 5XFAD mice and in wild-type sections treated with Aβ1-42 compared with wild-type controls. Pharmacological ablation of astrocytes resulted in a further 47% decrease in the spine size only in slices from 5XFAD mice and not in wild-type slices incubated with synthetic Aβ (Fig. 5). These results are in agreement with the reductions on synaptic density observed in our previous publication, using models of genetic ablation of astrocytes [17].

## Discussion

In the past few years, several studies have analyzed the role of astrocytes in vivo using animal models of AD. The results of the effects of ablation of astrocytes on amyloid pathology have been sometimes contradictory, depending on the approach and the animal model used. The first reports from 2013 from Kraft et al. [29] suggested that the genetic depletion of GFAP and vimentin in APP/PS1 mice resulted in increased amyloid plaque deposition, while in a different study GFAP and vimentin deficiency did not lead to differences in amyloid load [30]. In contrast, reduction of astrocytic activation by viral injection of the Gfa2–VIVIT construct [31] decreased Aβ pathology in APP/PS1 transgenic mice. More recently, we have reported that the ablation of reactive astrocytes in double transgenic APP23/GFAP-TK mice significantly augmented the levels of monomeric Aβ in brain homogenates, without affecting the plaque load or the number of plaques [17]. Therefore, it seems that the consensus is that the reduction in astrocyte density leads to higher amyloid levels, without changes in its aggregation or deposition. These results are in line with the data obtained in the present study, showing that pharmacological ablation of astrocytes leads to higher Aβ levels.

Regarding the molecular mechanism involved in the changes in amyloid pathology, the results from our studies suggest that astrocytes are involved in mechanisms of amyloid degradation and clearance, rather than affecting amyloid generation. We have demonstrated, using two different approaches, that the expression of neprilysin was decreased following astrocytic loss ([17] and present study). The effect on Apo-E protein expression was only significant in wild-type sections by L-AAA treatment. In addition, we observed that Ide gene expression and IDE secreted in the media was reduced in OBCSs treated with L-AAA, potentially contributing to the stability of amyloid in the culture media. Neprilysin is mainly expressed in neurons [32] but can also be found in activated astrocytes and microglia [15], while IDE is produced and secreted by neurons, oligodendrocytes, and microglia in the brain [33], and ApoE is mainly produced in astrocytes but can be synthesized by microglia [15]. It is possible that indirectly, loss of astrocytes could affect the levels of neprilysin and IDE in other cell types. In addition, astrocytes are involved in the clearance of Aβ through the BBB via the AQP4 channels expressed in their end feet and are also able to phagocytose amyloid [6, 34, 35]. Interestingly, recent studies of single cell transcriptomics have shown that the APOE gene was repressed in Alzheimer’s disease oligodendrocyte precursor cells (OPC), oligodendrocyte, and astrocyte subclusters, while it was increased in the microglial AD subcluster [36, 37]. Therefore, all of these observations point out to a protective role of astrocytes in AD, particularly mediating amyloid degradation and clearance, which can be compromised in AD brain at later stages.

In this study, we also investigated the relationships between microglia and astrocytes in WT and transgenic OBCSs. We observed that microglia from transgenic cultures seemed to have a different phenotype, compared to WT sections, showing more amoeboid-like morphology and higher Il-1β levels and a reduction in TGF-β and MCP1, representative of a pro-inflammatory profile. Intriguingly, TNFα expression was reduced in media from 5XFAD sections compared with WT OBCSs; however, the role of this cytokine in AD is contentious, because it can protect neurons against amyloid toxicity or induce apoptosis [38]. We also show that L-AAA treatment did not influence the density of microglia, but led to a reactive phenotype, contributing to a pro-inflammatory environment. Our data indicate that the effect of L-AAA on cytokine and chemokine expression is different in WT and transgenic sections, suggesting that the phenotype of the astrocytes could be different in transgenic animals. Interestingly, L-AAA induced a reduction in cytokines and chemokines produced in astrocytes (such as TGF-β, MIP-1α, and MCP1 as well as ApoE) in organotypic cultures of wild-type mice, with no changes in transgenic slides. However, pro-inflammatory cytokine IL-6 produced by microglia and astrocytes was increased in both WT and transgenic slices, indicating the elevation on inflammatory cytokines following incubation with L-AAA may not be exclusively secondary to an increase in Aβ. Therefore, our results indicate that astrocytes are different in WT and transgenic mice and may contribute to the activation of other glial cells, affecting their morphology and inflammatory profile. This is in line with studies showing that astrocytes can trigger microglia activation and regulate their cellular functions through the release of cytokines, chemokines, and growth factors [39].

Similarly, in this report, we demonstrate the effect of pharmacological ablation of astrocytes on synaptic density, which contributes to the exacerbation of the spine density and size in transgenic 5XFAD sections. Treatment with synthetic Aβ42 in WT slices had no effect in spine density. Previous reports have looked at the effect of injections of fibrillar Aβ42 in rat brain and detected a reduction in synaptic spines [40]; however, the effect of synthetic Aβ might depend on the concentration of amyloid, the incubation time, and the presence of fibrillar or oligomeric amyloid [41]. In wild-type tissue, L-AAA treatment also seems to affect the number of dendritic spines in sections treated with synthetic Aβ, but not their size. In previous studies, it was shown that L-AAA affected the cellular and synaptic properties in the lamprey spinal cord, suggesting a role of astrocytes in network locomotor activity [42]. These results reinforce the function of astrocytes regulating synapsis in AD, shown by us and others [17, 43]. These effects could be a consequence of their implications on Aβ clearance, the production of anti-inflammatory mediators, and/or the potential release of neurotrophic factors and metabolites [3].

## Conclusions

In conclusion, the present report shows the effects of the elimination of all types of astrocytes in an ex vivo model of AD (in contrast with previous studies, targeting only GFAP positive astrocytes) and allows the comparison with wild-type animals. In fact, we show that the profile of astrocytes in WT animals seems to be different to that in transgenic mice. Our results support the role of astrocytes on the removal of Aβ and the regulation of neuroinflammation in AD. In addition, the effects of L-AAA on dendritic spine density and size suggest a neuroprotective role of astroglia on memory processes.

Although the use of organotypic cultures of brain sections and of a chemical (L-AAA) to remove astrocytes has its limitations, this approach has allowed us to use a straightforward technology to elucidate the functions of astrocytes on amyloid pathology, neuroinflammation, and synaptic density. In addition, it is a useful tool for future studies on AD and other neurodegenerative diseases, avoiding the use of large numbers of animals and reducing animal suffering. In the future, it would be interesting to determine whether there is an effect of L-AAA using slices from the adult brain, which would have the advantage of mature adult brain morphology, metabolism, and presence of tau or Aβ deposition surrounded by activated glia.

New therapeutic approaches for AD could be based on the neuroprotective role of astrocytes, using pharmacological tools enhancing astrocytic proliferation. These effects could be promising even at late stages of the disease, when the formation of new synapsis and neuroinflammation could be more important than their function in regulating amyloid levels.

## Supplementary information

**Additional file 1: Figure S1.** L-AAA reduces the density of astrocytes but does not affect the viability of the tissue. Representative images of (A-D) Aldehyde dehydrogenase 1A (Aldh1a1), (E-G) GFAP, (H-K) Propidium Iodide and (L-M) Aβ staining (6C3) staining in cortex of WT and 5XFAD OBCSs treated with vehicle or L-AAA for 24 or 48hrs. Scale bar = 100μM.

## Data Availability

Materials described in the manuscript, including all relevant raw data, will be freely available to any scientist wishing to use them for non-commercial purposes, without breaching participant confidentiality. Data will be available upon request. All data generated or analyzed during this study are included in this published article [and its supplementary information files].

## Abbreviations

Aβ: Amyloid beta
AD: Alzheimer’s disease
ANOVA: Analysis of variance
ApoE: Apolipoprotein E
APP: Amyloid precursor protein
AQP4: Aquaporin-4
BACE1: Beta-site APP cleaving enzyme 1
BBB: Blood-brain barrier
BSA: Bovine serum albumin
CTF: Carboxy-terminal fragment
ELISA: Enzyme-linked immunosorbent assay
FAD: Familial Alzheimer’s disease
GFP: Green fluorescent protein
HRP: Horseradish peroxidase
IDE: Insulin degrading enzyme
IL: Interleukin
L-AAA: L-alpha-aminoadipate acid
NEP: Neprilysin
OBCSs: Organotypic brain culture slices
PBS: Phosphate-buffered saline
PFA: Paraformaldehyde
PVDF: Polyvinylidene difluoride
RIPA: Radioimmunoprecipitation assay
ROS: Reactive oxygen species
SEM: Standard error of the mean
TBS: Tris-buffered saline
TNF: Tumour necrosis factor
TBST: Tris-buffered saline with Tween
WT: Wild-type
