# Supplementary figures and images for: Pharmacological ablation of astrocytes reduces Aβ degradation and synaptic connectivity in an ex vivo model of Alzheimer’s disease
Source: J Neuroinflammation. 2021 Mar 17;18:73. doi: 10.1186/s12974-021-02117-y (PMC7972219; doi:10.1186/s12974-021-02117-y)

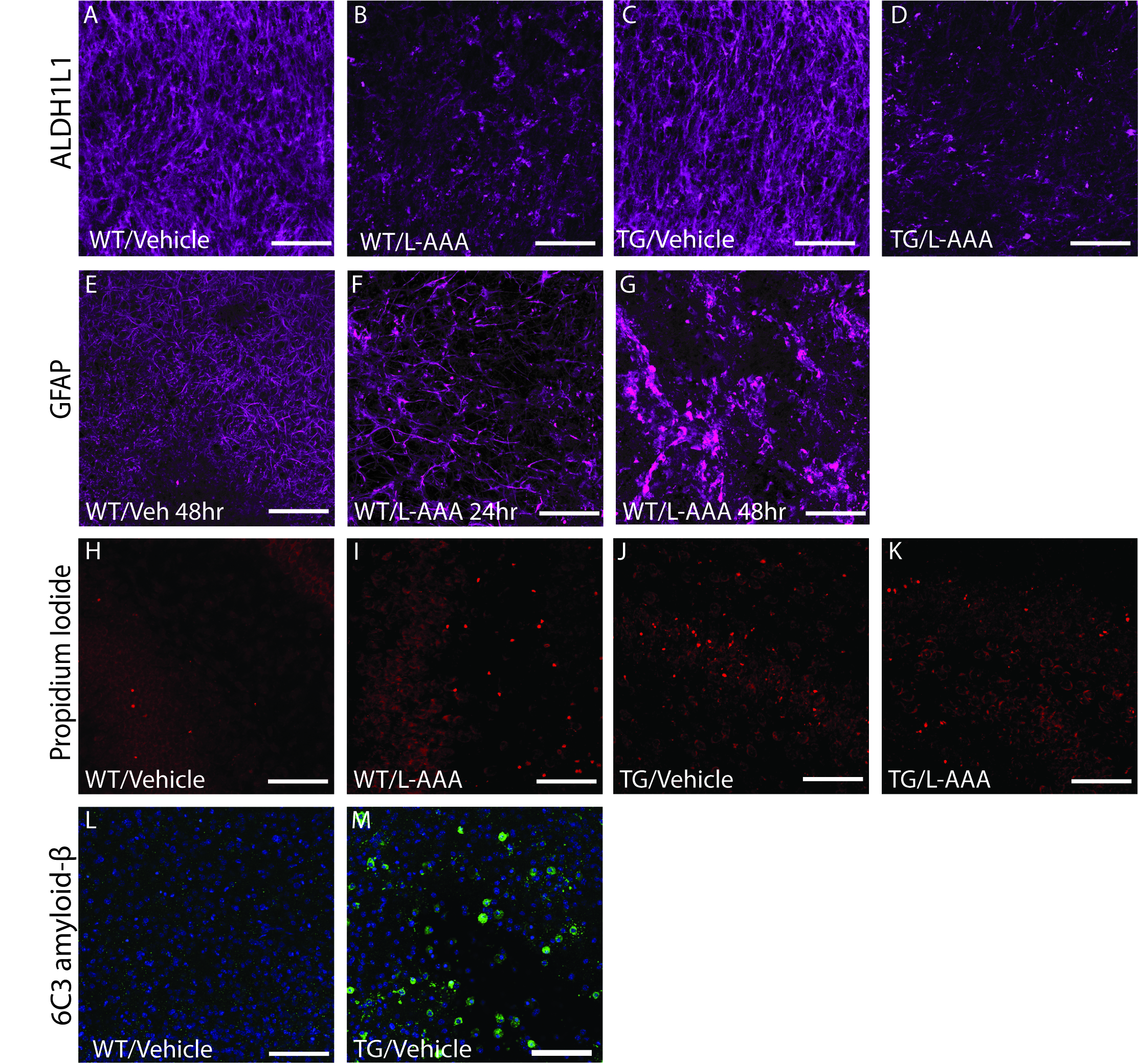

Supplement: Supplementary file 1 — Additional file 1: Figure S1. L-AAA reduces the density of astrocytes but does not affect the viability of the tissue. Representative images of (A-D) Aldehyde dehydrogenase 1A (Aldh1a1), (E-G) GFAP, (H-K) Propidium Iodide and (L-M) Aβ staining (6C3) staining in cortex of WT and 5XFAD OBCSs treated with vehicle or L-AAA for 24 or 48hrs. Scale bar = 100μM. [file 12974_2021_2117_MOESM1_ESM.tif]
